# Chronic shift‐lag promotes NK cell ageing and impairs immunosurveillance in mice by decreasing the expression of CD122

**DOI:** 10.1111/jcmm.16088

**Published:** 2020-11-13

**Authors:** Xiaokang Zeng, Caiying Liang, Jie Yao

**Affiliations:** ^1^ Medical Research Center and Department of Laboratory Medicine Shunde Hospital Southern Medical University (The First People's Hospital of Shunde, Foshan) Foshan China; ^2^ Guangdong Keyway Testing Technology Co, Ltd. Foshan China

**Keywords:** CD122, circadian rhythm disruption, Eomes, NK cell development, NK cell function

## Abstract

Long‐term subjection to shift work increases the risk of cancer. The purpose of the present study was to explore the mechanism by which chronic circadian disruption impairs natural killer (NK) cell immunosurveillance. Mice were subjected to light‐dark reverse every 4 days for 12 weeks to disrupt normal circadian rhythm. NK cell development and function were evaluated by flow cytometry. The mRNA and protein levels of period 1 (*per1*) and *per2* were suppressed, while circadian locomotor output cycle kaput (*CLOCK*) was increased in the shifted mice, indicating successful generation of the circadian rhythm disruption mouse model. Chronic shift‐lag promoted NK cell ageing, which is likely due to the reduction in *Ly49* family receptor expression in shifted NK. We further studied the effects of circadian rhythm disruption on NK cell function. Chronic shift‐lag inhibited NK cell secretion of granular CD107a and interferon gamma. Moreover, chronic shift‐lag attenuated the clearance of MHC‐I–deficient tumour cells by NK cells in vivo and promoted lung metastasis of B16F10 melanomas. Furthermore, chronic shift‐lag reduced NK cell killing function, which may be due to the suppression of Eomes transcription factor expression, which inhibiting the transcription of CD122. In conclusion, our findings suggest that chronic circadian disruption attenuates NK cell cytolytic activity by decreasing the expression of CD122.

## 1 INTRODUCTION

Natural killers (NKs) are a type of innate immune cell that can directly kill tumour cells without pre‐activation. It is well‐known that NK cell‐mediated immune monitoring not only kills tumour cells directly, but also indirectly releases cytokines to regulate other leucocytes such as macrophages, T cells and dendritic cells (DCs).^1^ NK cells can destroy infected and tumour cells by releasing cytolytic granules and interferon gamma (IFN‐γ). Among these molecular mechanisms, the release of granzyme B and perforin is the most effective way for NK cells to kill tumour cells.^2^ Another important function of NK cells is the release of pro‐inflammatory cytokines such as tumour necrosis factor‐alpha (TNF‐α).^3^ NK cells are derived from bone marrow and are tightly regulated by IL‐15 and its downstream signals; NK cell development in mice with full‐length IL‐15 or subunit deletion is severely damaged. IL‐15 signalling is extremely important for the survival and homeostasis of NK cells in peripheral immune niches.^4^

The transcription factors (TFs) of the T‐box family, T‐bet and Eomes, are the only T‐box proteins expressed in the immune system^5^ and broadly regulate leucocyte immune effects. Recent studies have documented the importance of T‐bet and Eomes in terms of NK cell development and function.^6^ Eomes is highly expressed in NK and memory CD8^+^ T cells, functioning to promote CD122 expression and maintain IL‐15 response. Eomes promotes NK cell development and maturation, and T‐bet is necessary for NK cell terminal maturation.^7^

Circadian rhythm (CR) is an endogenous autonomous rhythmic clock controlling the physiological activity of an organism to form a 24‐hour day‐night cycle. The major pacemaker of CR lies in the suprachiasmatic nucleus (SCN), which plays an important role in maintaining systemic CR by releasing endogenous regulatory factors.^8^ The molecular mechanism for maintaining circadian rhythm in the SCN and organs is regulated by feedback from the core circadian clock genes, including periods (*Per1‐3*), Cryptochrome (*Cry1* and *Cry2*) and *CLOCK*.^9^ Although the importance of circadian rhythm in the immune system has been recognized, little is known regarding CR in the regulation of NK cell function. NKs are innate immune cells important for combating cell deterioration and tumour metastasis. Organismal circadian disorders are likely to alter NK cell function. Some studies have reported that NK cell functions, including the release of cytokines and cytolytic factors, are strictly controlled by circadian rhythm.^10^

However, there is little evidence to prove the relationship between the disruption of circadian rhythm and NK cell development and function. Hence, the purpose of the present study was to investigate whether circadian rhythm disruption impairs NK cell function and explore the underlying mechanism. Under the shift‐lag schedule, circadian rhythm was examined by monitoring circadian genes. NK cell maintenance and development were evaluated under both resting and stimulating conditions. Finally, it was explored whether NK cell immunosurveillance impairment due to chronic shift‐lag induces circadian rhythm disruption; an RMA‐S tumour cell clearance assay and B16 melanoma lung metastasis assay were used to examine NK cell anti‐tumour function. We assumed that chronic shift‐lag would suppress NK cell cytolytic activity towards tumour cells by promoting NK cell ageing and down‐regulation of CD122.

## 2 MATERIALS AND METHODS

### 2.1 Animals, experimental design and animal ethics statement

The animal research protocols used in the present study were approved by the Animal Ethics Committee of Shunde Hospital, Southern Medical University. Male C57BL/6 mice (6‐8 weeks old) were purchased from Guangdong Medical Animal Center (Foshan, China) and housed in temperature‐controlled (22 ± 1°C) quarters on a 12/12 light‐dark (LD) cycle with access to water and food ad libitum. The control mice were kept under normal LD conditions, and the chronic shift‐lag mice were subjected to a reverse LD cycle every 4 days for 12 weeks.

### 2.2 Real‐time PCR

Total RNA was extracted from splenic NK cells obtained by flow sorting, from which cDNA was generated using a reverse transcription system (TAKARA). Quantitative real‐time polymerase chain reaction (RT‐qPCR) was performed on a BioRad CFX96 qPCR system using the SYBR ExScript PCR Kit (TAKARA). The primer sequences of the selected genes used in the present study are shown in Table 1. The relative expression levels were calculated using the 2^−∆∆CT^ method as previously described.^11^ The results were normalized to the mRNA expression level of *GAPDH*.

**Table 1:** Primer sequences for real‐time RT‐PCR

Name	Oligos	
GAPDH	F:CATCCACTGGTGCTGCCAAGGCTGT	R:ACAACCTGGTCCTCAGTGTAGCCCA
Per1	F:AACCATAGTACCCAGTTGTCGG	R:TCCTAAGCAACGCATATAGACCA
Per2	F: CCCTTGGGCTGTGTTAATAGTG	R: AACTTCTCGTACAAGCCTGGG
Per3	F:TCCCAACATTTTCACCACAACC	R:CCTGCAATGCTTCCGATGC
Bmal1	F:CGCCGCTCTCTGTTCTGTAG	R:GTGTCGAGAAACGTACTCCATAG
CLOCK	F:ATGGTGTTTACCGTAAGCTGTAG	R:CTCGCGTTACCAGGAAGCAT
CD107a	F: CAGCACTCTTTGAGGTGAAAAAC	R: ACGATCTGAGAACCATTCGCA
IFN‐γ	F: ATGAACGCTACACACTGCATC	R: CCATCCTTTTGCCAGTTCCTC

### 2.3 Small interfering RNA transfection

NK cells sorted from control or chronic shift‐lag mice were seeded onto 12‐well plates at a density of 2 × 10^5^/well in DMEM containing 10% foetal calf serum and 1000 U/mL IL‐2. The following day, cells were transfected with 25 nmol/L Per1 (s71484, Thermo Fisher) and Per2 (s71485, Thermo Fisher) small interfering RNA (siRNA) or 50 nmol/L negative control siRNA (4390844, Thermo Fisher) using Lipofectamine™ 3000 (Thermo Fisher). After 48 hours, the knockdown efficiency of Per1 and Per2 was examined by Western blotting.

### 2.4 Western blotting

NK cells from control or chronic shift‐lag mice were transfected with Per1‐siRNA and Per2‐siRNA or negative control siRNA. After 24 hours, the cells were pelleted and lysed in RIPA buffer (Beyotime). Total protein was isolated, and the concentration was estimated using a BCA assay (Beyotime). Equal amounts of protein (50 μg) were separated on 12% SDS‐PAGE gels (Beyotime) and transferred to PVDF membrane (Thermo Fisher). The membranes were blocked with 5% BSA for 2 hours at room temperature and subsequently incubated overnight at 4°C with the following specific antibodies: anti‐Per1 (1:1000, Abcam), anti‐Per2 (1:1000, Abcam), anti‐CLOCK (1:1000, Abcam) and anti‐β‐actin (1:1000, Beyotime). The following day, the membranes were washed and incubated with a secondary antibody (rabbit, 1:1000, Beyotime) for 2 hours at room temperature. Protein bands were visualized using ECL Western blotting substrate on a BioRad ChemiDoc MP system (BioRad, CA).

### 2.5 Flow cytometry

Flow cytometry analysis was carried out on a BD CANTO (BD Biosciences), and cell sorting was performed on a BD Aria II (BD Biosciences). Monoclonal antibodies against mouse CD3, NK1.1, NKp46, CD117, CD127, Ly49A, Ly49C/I, Ly49H, Ly49G2, NKG2D, NKG2A/C/E, CD11b, CD27, IFN‐g, CD107a, Annexin V, ki67 and isotype were purchased from eBioscience or Biolegend. For analysis of NK cell surface markers, antibodies were diluted in PBS containing 3% FBS. To analyse intracellularly expressed proteins, cells were fixed and subsequently permeabilized with Phosflow Perm buffer III (BD) and stained with the indicated antibodies.

### 2.6 Detection of apoptosis and proliferation of NK cells by FACS

Splenocytes were stained with antibodies against Nkp46, Annexin V and Ki67 (eBiosences). Fluorescence intensity was measured by FACS according to the manufacturer's instructions (BD Biosciences).

### 2.7 Cell proliferation assay

5(6)‐Carboxyfluorescein diacetate succinimidyl ester (CFSE) staining was used to analyse NK cell proliferation in vitro. Sorted NK cells were resuspended in DMEM containing 5% FBS and labelled with 10 μmol/L CFSE (Sigma) for 10 minutes at 37°C. Staining was quenched by the addition of 3 volumes of FBS for 1 minutes prior to washing the cells twice at 4°C with DMEM containing 5% FBS. The labelled cells were seeded on 24‐well plates (Corning Costar) and exposed to 1000 U/mL IL‐2. After 24 hours, cells were harvested and the intensity of CFSE staining was quantitated by FACS (BD Biosciences).

### 2.8 Detection of CD107a expression and intracellular staining of IFN‐γ

Each mouse was injected intraperitoneally with 200 µg poly I:C to activate NK cells. After 18 hours, poly I:C‐activated spleen cells (2 × 10^6^) were co‐cultured with the same number of RMA‐S and YAC‐1 tumour cells on 24‐well plates pre‐coated with an anti‐Ly49D antibody overnight at 4°C. To inhibit intracellular protein release, GolgiStop™ reagent (BD Biosciences) was added. Subsequently, an APC‐conjugated anti‐CD107a antibody, PE‐conjugated anti‐IFN‐γ or isotype was added. Medium only was used as a negative control. After 4 hours, cells were harvested for the detection of intracellular IFN‐γ and CD107a.^12^

### 2.9 In vivo RMA‐S clearance assay

Each mouse was injected intraperitoneally with 200 µg poly I:C to activate NK cells. After 18 hours, NK‐sensitive RMA‐S cells expressing GFP (10^6^) and non‐NK‐sensitive RMA cells expressing Ds‐Red (10^6^) were mixed and intraperitoneally injected into the pre‐activated mice. After a further 18 hours, the mice were killed. Tumour cells in the peritoneal cavity were washed out with PBS and collected. Flow cytometry was used to detect the percentages of RMA‐S and RMA cells.^13^

### 2.10 B16 melanoma lung metastasis mouse model

B16F10 melanoma cells were cultured to the log phase, resuspended in PBS, and 2 × 10^5^ cells were intravenously injected into each mouse. After 14 days, the mice were culled; the lungs were weighed, and the number of tumour nodules was counted. To clarify the role of NK cells, antibodies were used to eliminate NK cells in mice. Following B16 melanoma injection, each mouse was intraperitoneally injected with 1 mg PK136 antibody three times a week.

### 2.11 Blockade of CD122 with an anti‐CD122 monoclonal antibody

The control mice were kept under a normal LD cycle, and the chronic shift‐lag mice were subjected to a reverse LD cycle every 4 days for 12 weeks. During the final 2 weeks, the mice received 200 μg anti‐CD122 monoclonal antibody (TMβ1, Bio X Cell) intraperitoneally twice a week. The expression level of CD122 in NK cells was examined by FACS.

### 2.12 Statistical analysis

An unpaired Student's *t* test (two‐tailed) was performed using SPSS 22.0 (SPSS Inc, Chicago, IL, US). A *P*‐value <.05 was considered statistically significant. *P < .05, **P < .01 and ***P < .001. Data are expressed as the mean ± SD.

## 3 RESULTS

### 3.1 Chronic shift‐lag interferes with circadian gene expression and inhibits the expression of CD107a and IFN‐γ on NK cells

**FIGURE 1:**
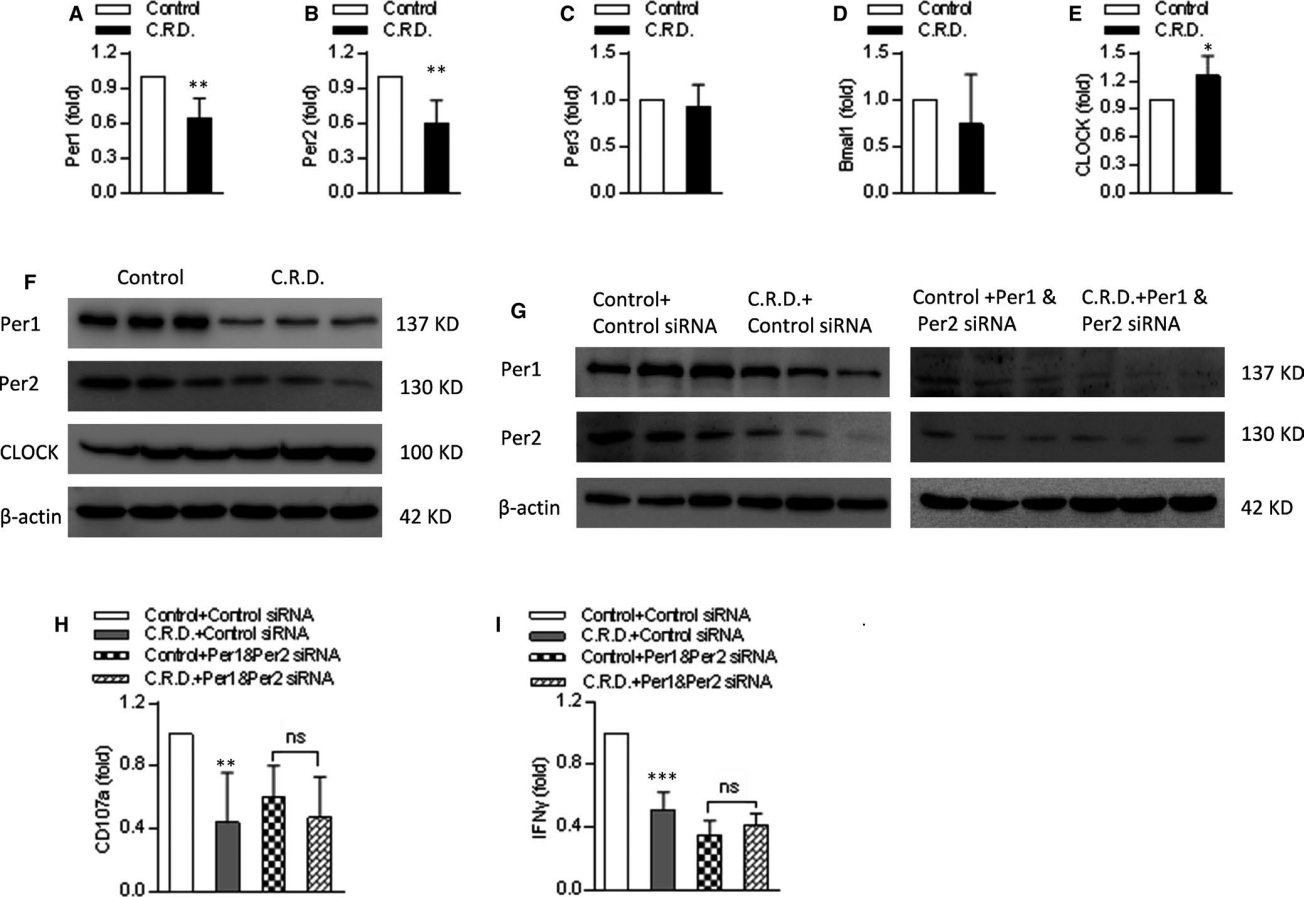
Chronic shift‐lag disrupts circadian rhythm and inhibits the expression of CD107a and IFN‐γ in NK cells. Splenic NK cells from control or chronic shift‐lag mice were sorted by flow cytometry. RT‐qPCR was used to analyse the mRNA levels of circadian rhythm genes such asper1 (A), per2 (B), per3 (C), Bmal1 (D) and CLOCK (E) in NK cells. The protein levels of per1, per2 and CLOCK were examined by Western blotting (F). Sorted NK cells were transfected with 25 nmol/L per1 and per2 siRNA or negative control siRNA and harvested after 48 h for protein analysis by Western blotting (G). The mRNA levels of CD107a and INF‐γ were detected by RT‐qPCR with or without Per1 and Per2 knockdown (H, I). Data are shown as the means ± SD. Unpaired Student's*t* tests (two‐tailed) were performed using the Prism software.*P‐*value of <.05 was considered significant. *P < .05, **P < .01

Circadian rhythm is regulated by a transcriptional‐translational feedback loop that involves a family of clock genes. The clock gene family includes aryl hydrocarbon receptor nuclear translocator‐like 1 (*Bmal1*), *CLOCK*, *per1*, *per2*, and cryptochrome (*cry1* and *cry2*). In addition, the orphan nuclear receptor *REV‐ERB* and *ROR* families are also reported to be feedback regulatory targets of *CLOCK*‐*Bmal1*.^14^ It is possible to change the circadian clock in mammals by changing the light period or food pattern; therefore, changing these signals can cause circadian clock disruption. To determine whether chronic shift‐lag disrupts circadian clock genes in splenic NK cells, we detected the mRNA levels of several circadian genes, such as *per1*, *per2*, *per3*, *Bmal1*, and *CLOCK*, in NK cells after chronic shift‐lag. In comparison with the control, chronic shift‐lag inhibited the mRNA expression of per1 and per2 and enhanced the mRNA expression of *CLOCK* (Figure 1A‐E). These results were consistent with the corresponding protein expression levels (Figure 1F). To verify the relationship between these gene changes and NK cell function, splenic NK cells were sorted from control and chronic shift‐lag mice and transfected with specific per1 and per2 siRNA. It was found that the expression levels of per1 and per2 in NK cells were significantly decreased following knockdown (Figure 1G). Further, the mRNA expression levels of CD107a and INF‐γ in NK cells were evaluated, and it was found that they were significantly decreased in NK cells from chronic shift‐lag mice (Figure 1H,I). We speculated that this may be the result of disruption of the circadian rhythm in NK cells by chronic shift‐lag. Therefore, we further evaluated the mRNA expression levels of CD107a and INF‐γ in NK cells following knockdown of per1 and per2, and it was found that they were decreased, with no significant difference between the control and chronic shift‐lag groups (Figure 1H,I). These results indicate that chronic shift‐lag disrupts the expression of NK cell circadian genes and reduces the mRNA levels of the NK cell function‐related genes CD107a and IFN‐γ.

### 3.2 Chronic shift‐lag slightly decreases the proportion and number of NK cells in the spleen and lungs

**FIGURE 2:**
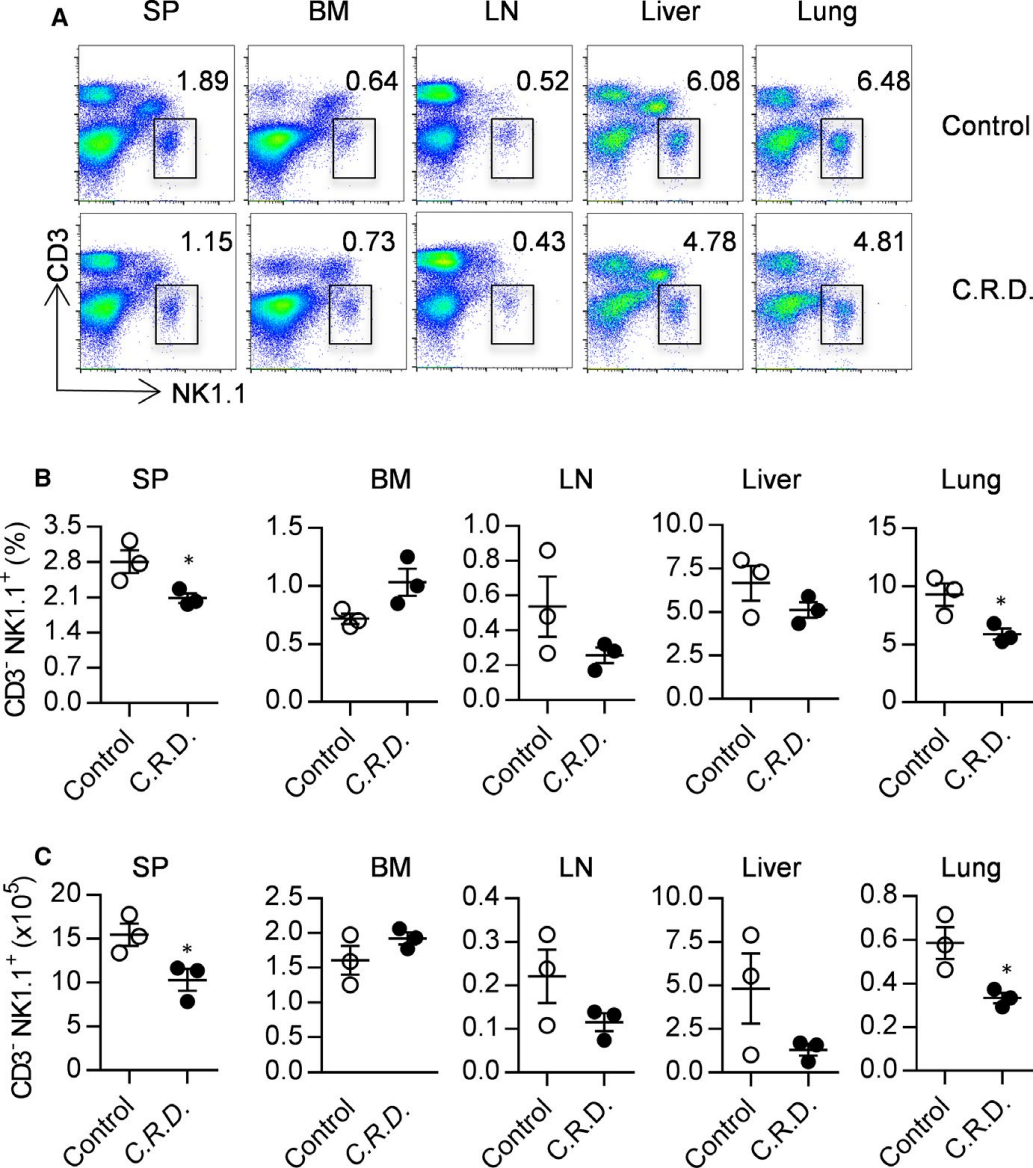
Shift‐lag induction reduces the number of NK cells in the spleen and lungs. Representative flow cytometry profiles of CD3^−^NK1.1^+^NK cells from the spleen (SP), bone marrow (BM), lymph nodes (LN), liver and lungs in control or chronic shift‐lag mice (A). Percentages of NK cells in the indicated organs and tissues from control and chronic shift‐lag mice were calculated (B). The absolute number of NK cells in the indicated organs and tissues from control and chronic shift‐lag mice were calculated (C). Each symbol indicates an individual mouse. Data represent at least three independent experiments. Data are shown as the means ± SD. Unpaired Student's*t* tests (two‐tailed) were performed using the Prism software. *P‐*value of <.05 was considered significant. *P < .05, **P < .01 and ***P < .001

Maintaining an appropriate number of NK cells in the resident organs is extremely important for immune monitoring^15^; hence, we wondered whether circadian rhythm disruption affects the physiology of NK cells. The proportion and absolute number of NK cells in different organs and tissues from mice subjected to chronic shift‐lag were detected by FACS. It was found that the proportion and number of NK cells in the spleen and lungs from chronic shift‐lag mice were decreased slightly but those in the bone marrow, lymph nodes and liver were unchanged. The proportion of NK cells in the spleen and lungs was decreased by 75% and 60%, respectively (Figure 2A,B), and the absolute number was decreased by 73% and 50%, respectively, after chronic shift‐lag (Figure 2C). These results suggest that circadian rhythm disruption may reduce the number of resident NK cells in the spleen and lungs, impairing NK cell function.

### 3.3 Chronic shift‐lag inhibits NK cell proliferation, promotes apoptosis and decreases activation in the spleen

**FIGURE 3:**
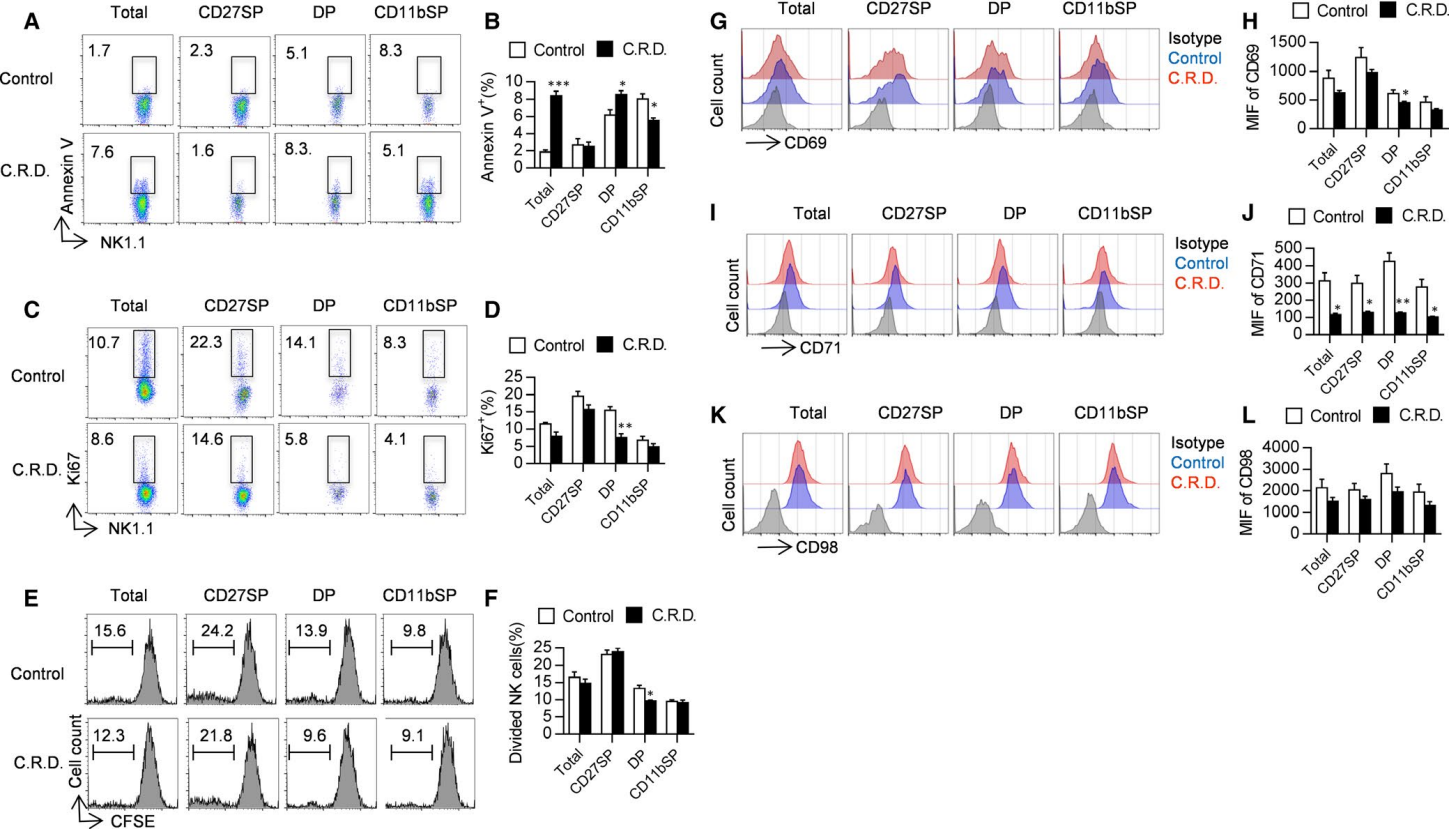
Shift‐lag induction promotes NK cell apoptosis, inhibits proliferation and decreases activation in the spleen. Expression of the apoptosis marker Annexin V in total NK cells and splenic NK cells at different stages from control and chronic shift‐lag mice was assessed by flow cytometry (A). Percentages of Annexin V^+^NK cells were calculated (B). Expression of the proliferation marker Ki67 in total NK cells and splenic NK cells at different stages from control and chronic shift‐lag mice was assessed by intracellular flow cytometry (C). Percentages of Annexin V^+^NK cells were calculated (D). Total NK cells and splenic NK cells at different stages from control and chronic shift‐lag mice were sorted by FACS. NK cells were stained with 10 μmol/L CFSE and subsequently cultured with 1000 U/mL IL‐2 for 24 h. The fluorescence intensity of CFSE was examined by FACS (E, F). Expression of the activation marker CD69 in total NK cells and splenic NK cells at different stages from control and chronic shift‐lag mice was assessed (G). The mean fluorescence intensity of CD69 in NK cells was calculated (H). Expression levels of the nutrition markers CD71 and CD98 in total NK cells and splenic NK cells at different stages from control and chronic shift‐lag mice were assessed (I, J). The mean fluorescence intensities of CD71 and CD98 in NK cells were calculated (K, L). All the data represent at least three independent experiments. Data are shown as the means ± SD. Unpaired Student's *t*tests (two‐tailed) were performed using the Prism software. *P‐*value of <.05 was considered significant. *P < .05, **P < .01

Next, we explored why circadian rhythm disruption reduces NK cell number in mice. We focused on the two major pathways that affect NK cell homeostasis: cell proliferation and apoptosis. Apoptotic NK cells were labelled with Annexin V and detected by FACs, and it was found that apoptosis of total NK cells in the spleen was significantly increased following chronic shift‐lag. Apoptosis of effective mature NK cells (CD27^+^CD11b^+^) was also enhanced after chronic shift‐lag. On the other hand, apoptosis of ageing NK cells (CD27^−^CD11b^+^) was inhibited following chronic shift‐lag (Figure 3A,B). These results indicate that the reduction in the number of total and effective NK cells was partly due to enhancement of cell apoptosis. Moreover, cell proliferation is important for maintaining the NK cell pool in the spleen; hence, we evaluated the proliferation of NK cells by labelling with the specific marker ki67 and detecting them by FACS. It was found that chronic shift‐lag did not affect total NK cell proliferation. Subsequently, we examined the proliferation of NK cells at different stages. These data show that except for ki67^+^CD27^+^CD11b^+^ NK cells, which were decreased by approximately 2.5‐fold, ki67^+^ cells at other stages were not changed following chronic shift‐lag (Figure 3C,D). We further verified the proliferation of NK cells in vitro. The total NK cells and the proportion of NK cells at different stages from control mice and chronic shift‐lag mice were sorted and stained with CFSE, followed by the addition of 1000 U/mL IL‐2 for 24 hours. The CFSE fluorescence intensity of NK cells was measured to assess their proliferation. The results are consistent with those of the in vivo experiments, where the proliferation of CD27^+^CD11b^+^ NK cells in chronic shift‐lag mice was significantly inhibited, while that of NK cells at other stages was unaffected (Figure 3E,F). This indicates that the number of NK cells in the spleen was decreased following chronic shift‐lag, likely due to the enhancement of NK cell apoptosis. Furthermore, we also examined the activation and nutritional status of NK cells, and it was found that the expression level of the activation marker CD69 was decreased on NK cells from chronic shift‐lag mice (Figure 3G,H), suggesting that chronic shift‐lag inhibits NK cell activation. In addition, we examined the nutritional status of NK cells by detecting the nutrition markers CD71 and CD98 (Figure 3I‐L), and it was found that the nutritional level of NK cells was low following chronic shift‐lag, which may also lead to reduced cell function.

### 3.4 Chronic shift‐lag promotes NK cell ageing in the mouse spleen

**FIGURE 4:**
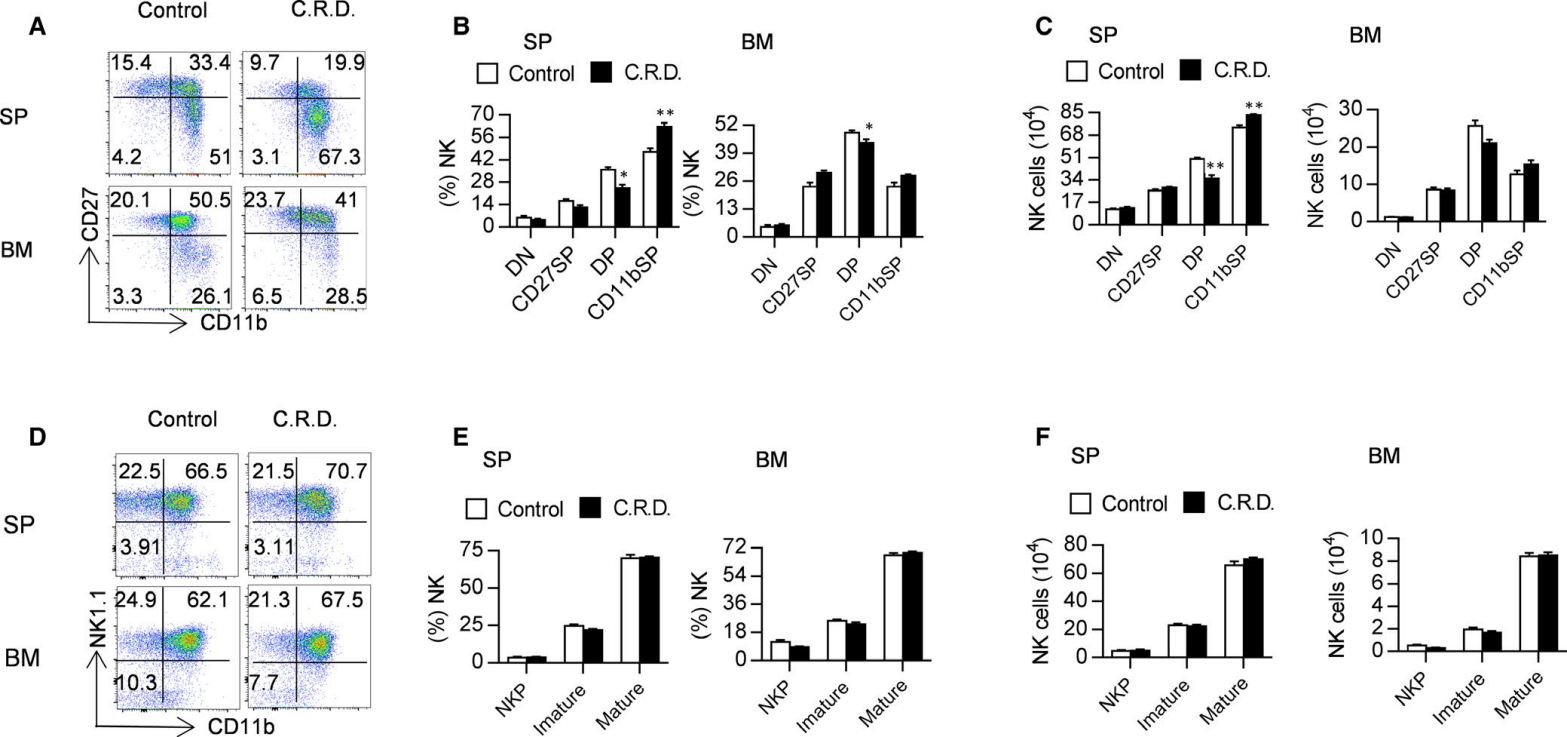
Shift‐lag induction promotes NK cell ageing in the spleen and bone marrow. Representative flow cytometry profiles (A), percentage (B) and absolute number (C) of NK cell subsets: DN (CD27^−^CD11b^−^), CD27SP (CD27^+^CD11b^−^), DP (CD27^+^CD11b^+^) and CD11bSP (CD27^−^CD11b^+^) in the spleen and bone marrow from control and chronic shift‐lag mice. Representative flow cytometry plots (D), percentage (E) and absolute number (F) of NKp (NK1.1^−^CD11b^−^), imNK (NK1.1^+^CD11b^−^) and mNK (NK1.1^+^CD11b^+^) cells gated on CD3^−^CD122^+^splenocytes and bone marrow cells from control and chronic shift‐lag mice. All the data represent at least three independent experiments. Data are shown as the means ± SD. Unpaired Student's*t* tests (two‐tailed) were performed using the Prism software. *P‐*value of <.05 was considered significant. *P < .05, **P < .01

NK cells can be divided into different developmental stages including NK cell precursors (NKp), immature NK cells (iNK) and mature NK cells (mNK). To understand how chronic shift‐lag affects NK cell development, we first quantitated the number of NK cells at different stages of development, including NKp, iNK and mNK. These data show that the proportion of effective mNK cells (CD27^+^ CD11b^+^) was decreased in the spleen and bone marrow following chronic shift‐lag, while the number of ageing NK cells (CD27^−^CD11b^+^) was increased in the spleen. The proportion of functional NK cells (CD27^+^CD11b^+^) was reduced by approximately 1.68‐fold in the spleen and 1.23‐fold in the bone marrow following chronic shift‐lag, while the proportion of ageing NK cells (CD27^−^CD11b^+^) was increased by approximately 1.32‐fold in the spleen but did not change in the bone marrow (Figure 4A,B). The number of effective mNK cells was reduced and that of ageing NK cells was increased in the spleen (Figure 4C). Subsequently, we examined the number of NK cells at different stages using the markers NK1.1 and CD11b, and it was found that the proportion and absolute number of NKp (NK1.1^+^CD11b^+^), iNK (NK1.1^+^CD11b^−^), and mNK (NK1.1^+^CD11b^+^) in the spleen and bone marrow were not significantly changed following chronic shift‐lag (Figure 4D‐F). These results indicate that disruption of circadian rhythm by chronic shift‐lag induction may promote NK cell ageing in the spleen.

### 3.5 Chronic shift‐lag promotes the expression of immature receptors and inhibits the expression of Ly49 family receptors on NK cells in the spleen and bone marrow

**FIGURE 5:**
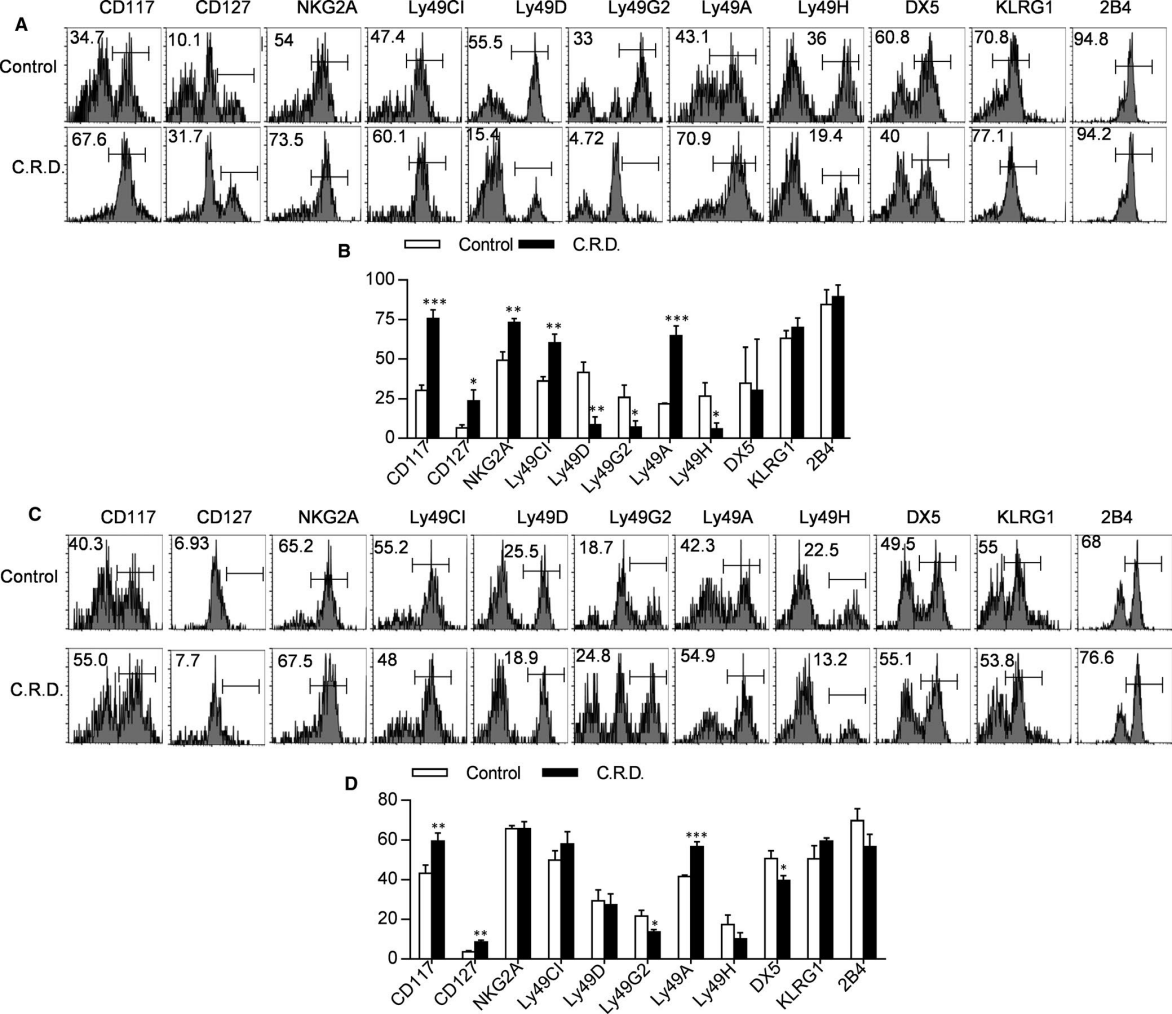
Shift‐lag induction promotes the expression of immature receptors and inhibits the expression of Ly49 family receptors on NK cells from the spleen and bone marrow. Flow cytometry analysis of development‐related NK cell receptors on CD3^−^NK1.1^+^cells in the spleen from control and chronic shift‐lag mice (A). Numbers indicate the percentage of receptor‐positive NK cells in the spleen (B). Flow cytometry analysis of development‐related NK cell receptors on CD3^−^NK1.1^+^cells in the bone marrow from control and chronic shift‐lag mice (C). The numbers indicate the percentage of receptor‐positive NK cells in the bone marrow (D). All the data represent at least three independent experiments. Data are shown as the means ± SD. Unpaired Student's*t* tests (two‐tailed) were performed using the Prism software. *P‐*value of <.05 was considered significant. *P < .05, **P < .01 and ***P < .001

NK cells recognize tumour cells by activating and inhibiting receptors on the cell surface.^16^ Ly49 family receptors can interact with MHC‐I molecules to activate or inhibit NK cells. Normal cells express MHC‐I, which interacts with Ly49 family receptors to transmit inhibitory signals to NK cells, while aberrant cells expressing a low level of MHC‐I transmit an activation signal for killing.^17^ We analysed the expression of receptors on the surface of NK cells in the spleen and bone marrow from chronic shift‐lag mice. The results show that the expression levels of several Ly49 family members (including Ly49 D, G and H) were reduced in chronic shift‐lag mice. In contrast, the expression levels of immature receptors (CD117 or CD127) were increased in chronic shift‐lag mice (Figure 5A‐D). These results indicate that the abnormal development of NK cells in chronic shift‐lag mice may be caused by the abnormal expression of surface receptors. The expression of Ly49 family receptors was inhibited in chronic shift‐lag mice, suggesting that the immunosurveillance ability of NK cells was likely damaged.

### 3.6 Chronic shift‐lag impairs NK cell‐mediated immunosurveillance

**FIGURE 6:**
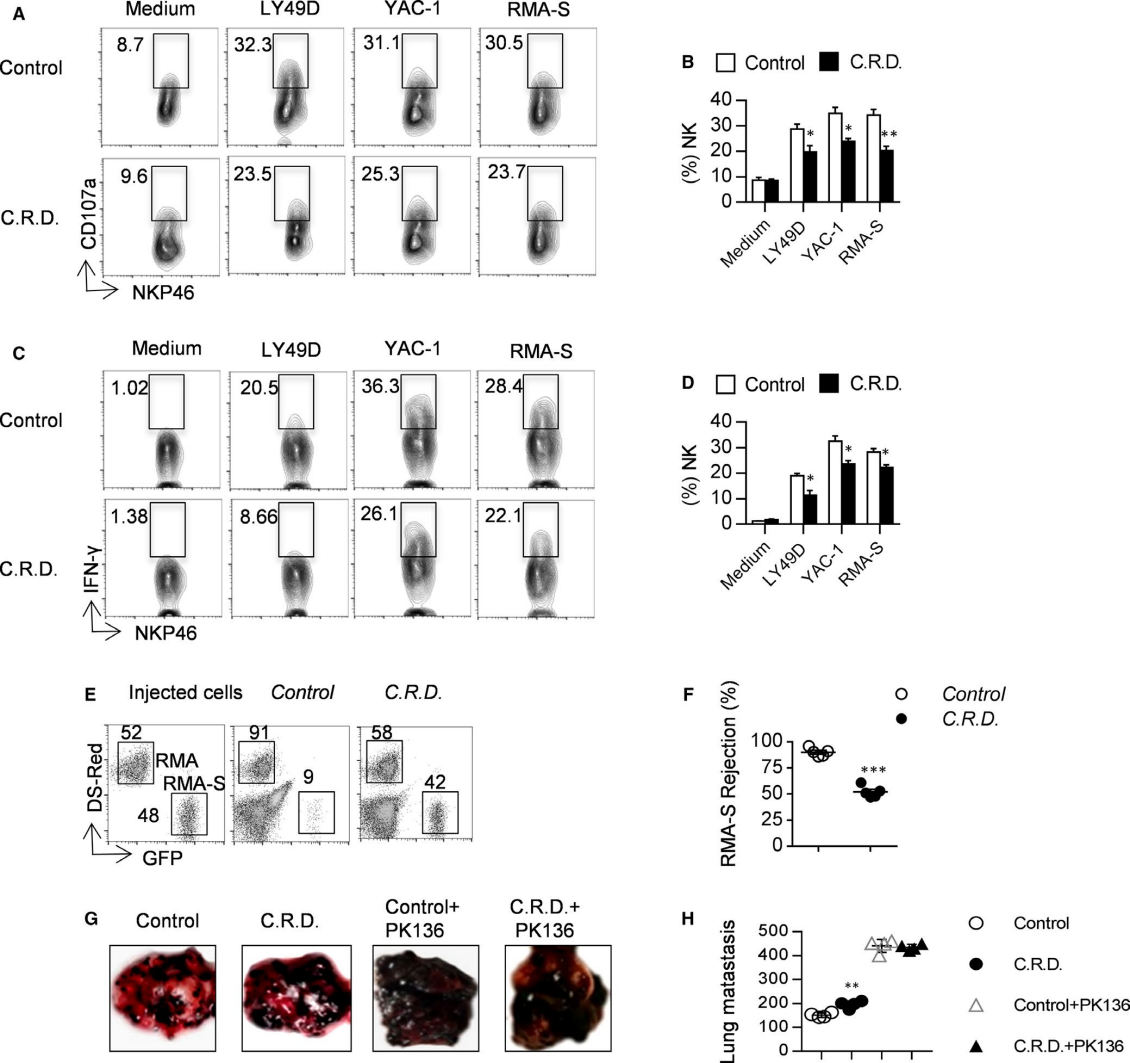
Shift‐lag induction impairs NK cell‐mediated immunosurveillance. Representative flow cytometry profiles and percentages of CD107a (A, B) and IFN‐γ (C, D) expression on poly I:C‐activated splenic NK cells stimulated by an anti‐Ly49D antibody or YAC‐1 or RMA‐S tumour cells. Representative flow cytometry plot of RMA‐S cells in the peritoneal cavity 18 h after injection of the indicated mice with a mixture of NK cell‐sensitive RMA‐S cells expressing GFP and non‐NK cell‐sensitive RMA cells expressing DsRed (E). Percentages of rejected RMA‐S cells were calculated (F). For the B16 metastasis assay, the indicated mice pretreated with or without an anti‐NK1.1 antibody were intravenously injected with 2 × 10^5^B16 cells. After 14 d, the number of tumour nodules was counted (G, H). Each symbol represents an individual mouse. All the data represent at least three independent experiments. Data are shown as the means ± SD. Unpaired Student's*t* tests (two‐tailed) were performed using the Prism software. *P‐*value of <.05 was considered significant. *P < .05, **P < .01 and ***P < .001

Up‐regulation of CD107a on the surface of NK cells can increase the lysis of target cells; therefore, we evaluated the expression of CD107a in NK cells following stimulation in vitro. In comparison with the control, fewer NK cells from chronic shift‐lag mice had up‐regulated expression levels of CD107a following stimulation with RMA‐S or YAC‐1 cells. Interestingly, NK cells from chronic shift‐lag mice expressed CD107a at a lower level after stimulation with an anti‐Ly49D antibody (Figure 6A,B). Collectively, these data indicate that circadian rhythm disruption inhibits the expression of CD107a on NK cells, suggesting that NK cells from chronic shift‐lag mice likely display a reduced ability to release perforin.

NK cells secrete IFN‐γ by either activating receptors that bind ligands on tumour cells or priming with antibodies such as anti‐Ly49D; thus, we analysed the critical role of chronic shift‐lag in IFN‐γ production by NK cells. NK cells were co‐cultured with hematopoietic tumour cells, RMA‐S and YAC‐1, or stimulated with an anti‐Ly49D antibody. NK cell secretion of IFN‐γ increased after different stimulation; however, in chronic shift‐lag mice, this effect was almost completely abolished (Figure 6C,D); hence, circadian rhythm may be an important element in the promotion of IFN‐γ production by NK cells.

NK cells are the main defence against allogeneic bone marrow and tumour cells. We further examined the ability of NK cells to clear RMA‐S tumour cells in vivo and found that rejection in chronic shift‐lag mice was lower than that in control mice (Figure 6E,F). Lastly, we investigated whether chronic shift‐lag impairs the ability of NK cells to prevent metastasis of B16 melanoma. Notably, B16 cell metastases in the lungs of chronic shift‐lag mice were more numerous than those in the lungs of control mice. However, after clearing of NK cells by NK1.1 antibody (PK136), the lung metastasis rate of B16 melanoma in the control group and the chronic shift‐lag group increased significantly but there was no significant difference between them, indicating that NK cells play an important role in inhibiting lung metastasis of B16 melanoma (Figure 6G,H). These data suggest that chronic shift‐lag disrupts NK cell function by inhibiting the release of perforin and IFN‐γ; hence, chronic shift‐lag may reduce NK cell immunosurveillance.

### 3.7 Chronic shift‐lag disturbs the expression of T‐bet, Eomes and CD122 on NK cells

**FIGURE 7:**
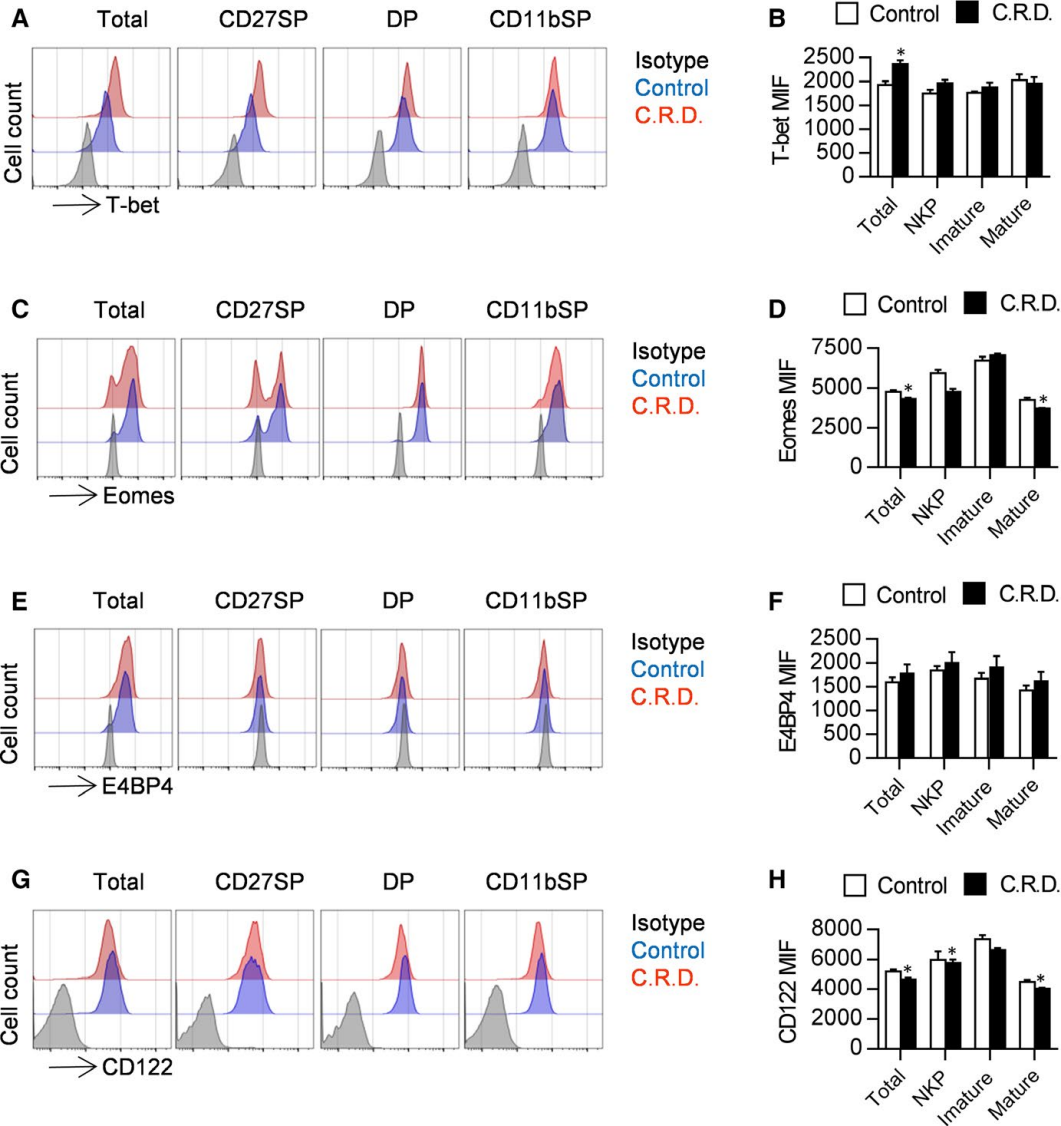
Shift‐lag induction disturbs T‐bet, Eomes and CD122 expression in NK cells. Representative flow cytometry profiles and mean fluorescence intensity of T‐bet in total NK cells and splenic NK cells at different stages from control or chronic shift‐lag mice (A, B). Representative flow cytometry profiles and mean fluorescence intensity of Eomes in total NK cells and splenic NK cells at different stages from control or chronic shift‐lag mice (C, D). Representative flow cytometry profiles and mean fluorescence intensity of E4BP4 in total NK cells and splenic NK cells at different stages from control or chronic shift‐lagmice (E, F). Representative flow cytometry profiles and mean fluorescence intensity of CD122 in total NK cells and splenic NK cells at different stages from control or chronic shift‐lag mice (G, H). All the data represent at least three independent experiments. Data are shown as the means ± SD. Unpaired Student's*t* tests (two‐tailed) were performed using the Prism software. *P‐*value of <.05 was considered significant. *P < .05

Abnormal metabolism causes aberrant expression of transcription factors and interferes with T cell differentiation^18^; therefore, we wondered whether circadian rhythm disruption affects the expression of transcription factors that mediate NK cell function. T‐bet, Eomes and E4BP4 are important transcription factors expressed during NK cell maturation; thus, we evaluated their protein expression levels by flow cytometry. The results show that the expression of T‐bet in NK cells from chronic shift‐lag mice was significantly increased, while that of Eomes was decreased (Figure 7A‐D). These results are consistent with those of previous studies that found T‐bet and Eomes to be antagonistic in NK cells. Next, we further tested the key transcription factor E4BP4, which determines the survival of NK cells, and it was found that the expression of E4BP4 in NK cells from chronic shift‐lag mice was normal (Figure 7E,F). Eomes plays an important role in NK cell maturation by regulating CD122 expression; therefore, we evaluated the level of CD122 expression inNK cells from chronic shift‐lag mice. The results show that the level of CD122 in NK cells from chronic shift‐lag mice was consistent with that of Eomes, both of which were significantly reduced (Figure 7G‐H). Therefore, chronic shift‐lag disrupts the balance between T‐bet and Eomes in NK cells, reducing CD122 expression, which weakens the response of NK cells to IL‐15.

### 3.8 Blockade of CD122 decreases the secretion of CD107a and INF‐γ from NK cells

**FIGURE 8:**
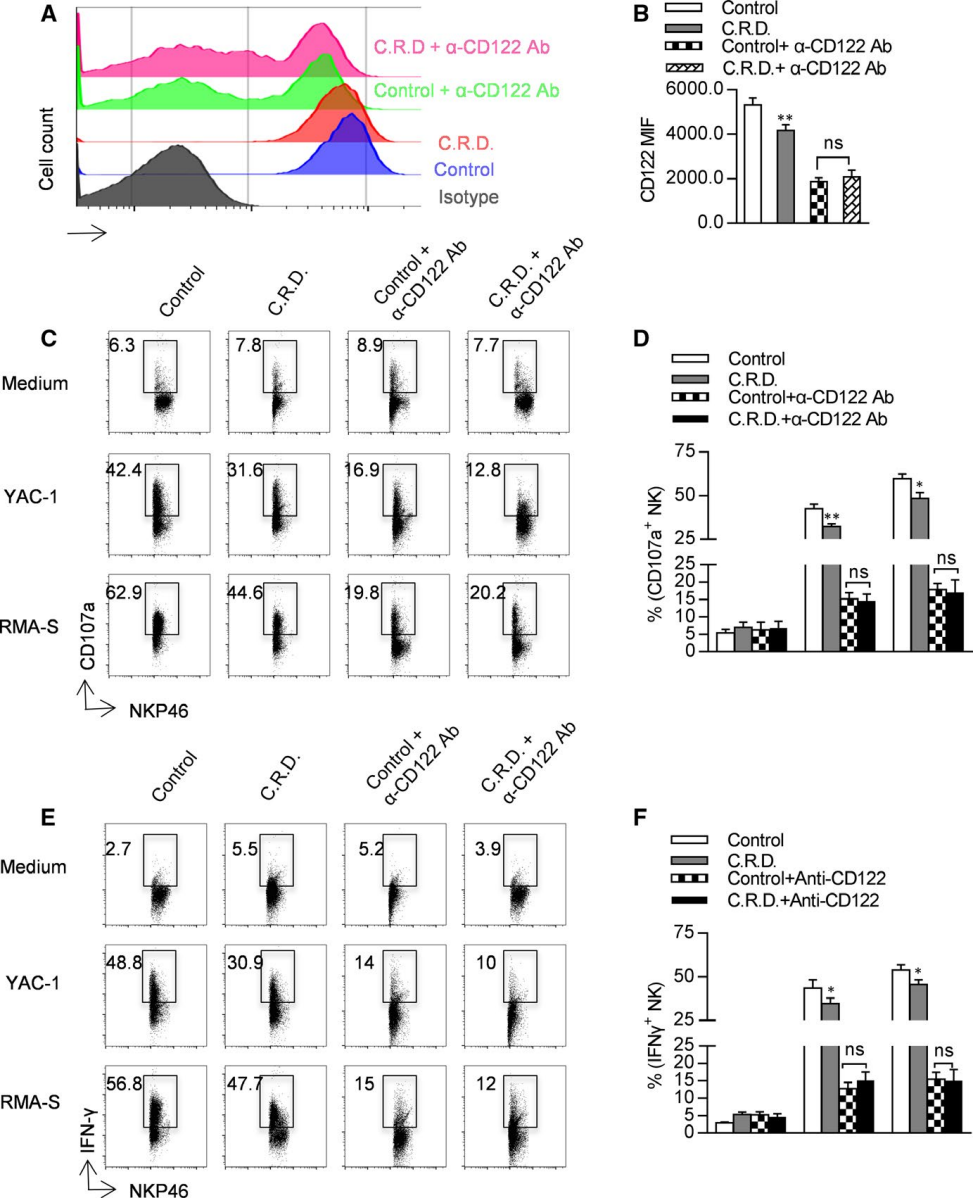
Blockade of CD122 decreases the secretion of CD107a and INF‐γ from NK cells. A chronic shift‐lag mouse model was established, and an anti‐CD122 monoclonal antibody was intraperitoneally injected during the final 14 d. Flow cytometry was used to detect the expression of CD122, and the mean fluorescence intensity was calculated (A, B). Splenic NK cells were stimulated with RMAS or YAC1 to detect the level of secreted CD107a (C, D). Splenic NK cells were stimulated with RMAS or YAC1 to detect the level of secreted INF‐γ (E, F). All the data represent at least three independent experiments. Data are shown as the means ± SD. Unpaired Student's*t* tests (two‐tailed) were performed using the Prism software. *P‐*value of <.05 was considered significant. *P < .05, **P < .01

To verify that chronic shift‐lag damages NK cell function by inhibiting CD122, we used an anti‐CD122 monoclonal antibody to block CD122 during the final 2 weeks of chronic shift‐lag mouse model establishment. Following completion of the model, CD122 expression on NK cells was examined, and it was found that anti‐CD122 antibody treatment significantly reduced the expression of CD122 on NK cells (Figure 8A,B). We further explored the effect of CD122 blockade on the function of NK cells by evaluating their ability to secrete CD107a and IFN‐γ following stimulation with tumour cells. It was found that after blocking CD122, the ability of NK cells to secrete CD107a and IFN‐γ was significantly reduced; however, interestingly, there was no significant difference between the control and chronic shift‐lag groups (Figure 8C‐F). These results indicate that blockade of CD122 inhibits NK cell function and further suggests that chronic shift‐lag impairs NK cell function by inhibiting CD122 expression.

## 4 DISCUSSION

Circadian rhythm regulates many physiological processes including immune cell development and function.^19^ The SCN is the centra lcontroller of the circadian rhythm and regulates the peripheral clock through the action of hormones or other signals.^20^ For example, immune tissue receives rhythmic input, thereby regulating clock gene expression in the immune system including NK cells.^10^, ^21^ NK cell function appears to be regulated by circadian rhythm, such as clock genes or cytokines.^22^ The present study suggests that circadian rhythm plays an important role in the development and functional acquisition of NK cells. During circadian disturbances, the ability of NK cells to secrete IFN‐γ and CD107a was significantly limited. The immunosurveillance function of NK cells with a disrupted circadian rhythm was decreased, manifesting as a reduction in the clearance of MHC‐I‐deficient tumour cells and a decreased ability to clear B16 melanoma cells. With respect to the underlying mechanism, we found that senescent NK cells in chronic shift‐lag mice were significantly increased, while the expression levels of Eomes and CD122 in NK cells were decreased. We speculated that the insensitivity of chronic shift‐lag NK cells to IL‐15 signals may be the main reason for the reduction in immunosurveillance.

We constructed a chronic shift‐lag mouse model by subjecting mice to a reverse light‐dark cycle every 4 days for 12 weeks.^23^ Splenic NK cells were sorted by flow cytometry. Certain important molecules that regulate circadian rhythm, such as per1‐3, Bmal1 and CLOCK, were examined by RT‐qPCR and Western blotting. These data show that chronic shift‐lag causes the abnormal expression of molecular clock genes in NK cells, which is in accordance with the findings reported by Ryan et al^24^, ^25^ demonstrating that chronic shift‐lag caused the abnormal expression of *per2* and *Bmal1* in NK cells. We further explored the relationship between chronic shift‐lag and NK cell function, and found that chronic shift‐lag inhibits the expression of CD107a and IFN‐γ in NK cells, while knockdown of per1 and per2 abolishes this inhibitory effect. Logan et al^26^ found that per1 deficiency significantly inhibits the mRNA expression levels of perforin and INF‐γ in splenic NK cells. Moreover, Liu et al^27^ found that splenic NK cells from per2‐knockout mice secrete less IFN‐γ after LPS stimulation. Our results are consistent with their reports. These data indicate that chronic shift‐lag disturbs the NK cell clock and inhibits NK cell function; therefore, we further studied the effect of chronic shift‐lag on NK cells.

Normal circadian rhythm is extremely important for the immune system. NK, as essential immunosurveillance cells, can cause disease when functioning abnormally^21^; therefore, we were curious as to whether the disruption of circadian rhythm impairs the number and function of NK cells. Our data show that the proportion and absolute number of NK cells in the spleen and lungs of chronic shift‐lag mice are reduced, indicating that circadian disturbances affect the number of NK cells, which may be due to the promotion of NK cell apoptosis. Studies have shown that chronic shift‐lag accelerates body ageing.^28^ It is speculated that circadian rhythm disorder promotes NK cell apoptosis. In addition, circadian disorders also inhibit the activation of NK cells and reduce the expression of the nutrition receptor CD71, indicating that circadian disorders can also inhibit NK cell activation and reduce nutritional levels. It is also speculated that circadian rhythm disorder may disrupt the development of NK cells and impair immunosurveillance.

The normal development of NK cells is critical to their function. We found that NK cells developed abnormally in chronic shift‐lag mice. Ageing NK cells (CD27^−^CD11b^+^) were significantly increased in both bone marrow and spleen, while functional NK cells (CD27^+^CD11b^+^) were significantly decreased in chronic shift‐lag mice, suggesting that circadian rhythm disorder may promote ageing of NK cells and affect their function. NK cells express different receptors at different developmental stages, and we found that circadian disturbances disrupt the expression of NK cell receptors. Following disruption of the circadian rhythm, NK cells have increased expression of certain immature receptors such as CD117 and CD127, while the expression of other functional receptors was reduced, such as Ly49D, Ly49G2 and Ly49H, suggesting that circadian rhythm disorder inhibits functional receptor expression and causes abnormal development of NK cells.

Since the number of functional NK cells is decreased in chronic shift‐lag mice, we wondered whether chronic shift‐lag impairs the function of NK cells. We found that secretion of CD107a by NK cells with circadian rhythm disorder is significantly reduced irrespective of stimulation with an anti‐Ly49D antibody or RMA‐S/YAC‐1 tumour cells. In addition, secretion of IFN‐γ by NK cells with circadian rhythm disorder is also reduced, indicating that circadian rhythm disruption decreases the immunosurveillance ability of NK cells. We further verified the function of NK cells by evaluating the rejection of MHC‐I‐deficient tumour cells in vivo. The rejection of RMA‐S cells is reduced in mice possessing NK cells with circadian rhythm disruption. We further analysed whether circadian rhythm disruption affects metastasis of B16 melanoma cells, and it was found that lung metastasis of B16 cells was aggravated in mice possessing NK cells with circadian rhythm disruption according to lung weight and number of tumour colonies. Moreover, Eomes, E4BP4 and T‐bet are important transcription factors that regulate NK cell development and function.^29^ The expression of T‐bet in NK cells with circadian rhythm disruption was significantly reduced, while the expression of Eomes was significantly increased. Eomes regulates NK cell development and function by transcriptionally regulating CD122. CD122, the gamma chain of the IL‐15 receptor, plays a key role in maintaining IL‐15 stimulation.^30^ CD122 expression on NK cells from chronic shift‐lag mice was significantly reduced. Following blockade of CD122 with a specific antibody, the secretion of CD107a and IFN‐γ by NK cells was decreased. Interestingly, following blockade of CD122, the effect of chronic shift‐lag on the inhibition of CD107a and IFN‐γ secretion from NK cells disappeared. Yuan et al^31^ found that blocking CD122 with an antibody can inhibit IFN‐γ secretion by T cells, alleviating autoimmune type I diabetes. Richmond et al^32^ demonstrated that blocking CD122 can reverse vitiligo in mice, which may be a result of inhibiting the production of INF‐γ in resident memory T cells. Our results suggest that chronic shift‐lag inhibits NK cell function by down‐regulating CD122. Further, stimulation of IL‐15 signalling is important for the maintenance of NK cell function; therefore, we speculate that circadian rhythm disruption leads to a decrease in NK cell immune monitoring ability by reducing the expression of Eomes and CD122, and ultimately reducing the response to IL‐15.

## 5 CONCLUSIONS

In conclusion, the present study demonstrates that circadian rhythm disruption slightly reduces the number of NK cells in the spleen and lungs. This process is achieved through the promotion of NK cell apoptosis and the inhibition of proliferation. Interestingly, circadian rhythm disruption promotes NK cell ageing, resulting in the accumulation of CD27^−^CD11b^+^ NK cells, which may be caused by inhibition of the expression of Ly49 family receptors. Circadian rhythm disruption reduces the ability of NK cells to release CD107a and IFN‐γ, clear MHC‐I‐deficient tumour cells, and inhibit lung metastasis of B16 melanoma, which is achieved through a reduction in the expression of CD122 on NK cells. Therefore, frequent shift work may damage NK cell‐mediated immunosurveillance and promote tumorigenesis.

## CONFLICT OF INTEREST

The authors declare that they have no competing interests.

## AUTHOR CONTRIBUTIONS

**Xiaokang Zeng:** Formal analysis (equal); investigation (equal). **Caiying Liang:** Data curation (equal); investigation (equal). **Jie Yao:** Supervision (equal); validation (equal); writing‐review & editing (equal).

## Data Availability

The data used to support the findings of this study are available from the corresponding author upon request.
